# Effects of innovation efficiency of high-tech industries on CO_2_ emissions in China: A spatial Durbin econometric analysis

**DOI:** 10.1371/journal.pone.0264017

**Published:** 2022-05-10

**Authors:** Lingyan Lou, Hongli Wang, Shen Zhong

**Affiliations:** 1 School of Finance, Harbin University of Commerce, Heilongjiang, PR China; 2 School of Economics and Management, Harbin Engineering University, Heilongjiang, PR China; Universidad Nacional Autonoma de Nicaragua Leon, NICARAGUA

## Abstract

As a responsible country, China should take on heavy responsibility of energy conservation and emission reduction, and it is an inevitable choice for China to develop high-tech industries and improve the innovation efficiency of high-tech industries in order to alleviate the current environmental pressure. Therefore, this paper takes the panel data of 30 provinces in China from 2005 to 2016 as the research sample, on the basis of using DEA Global-Malmquist index to measure the innovation efficiency of high-tech industries, it constructs three spatial weight matrices, by including spatial geography, spatial economic geography nesting and innovation, and then it uses Spatial Durbin Model to empirically analyze the effect of innovation efficiency of high-tech industry on CO_2_ emissions in China from spatial perspective. The results indicate: firstly, in China, CO_2_ emissions gradually increase from 2006 to 2012, however, some provinces have declined after 2012. And CO_2_ emissions present a descending trend from eastern coastal area to central and western region. Secondly, affected by “warning effect”, CO_2_ emissions show a significant negative spatial spillover effect. Thirdly, the overall level of innovation efficiency of high-tech industries in China is not high, and its impact on CO_2_ emissions is not a simple linear relationship, but shows an “inverted N–shaped” curvilinear relation, and its decomposition index EC and TC also have similar characteristics. Obviously, the research in this paper provides a necessary theoretical support for China and some emerging developing countries to rational formulating and effective implementing the energy conservation and emission reduction policies.

## Instruction

Since the 1760s, with the vigorous development of the industrial revolution, various countries in the world have significantly increase the demand for fossil fuels, such as oil and coal, and fossil fuels have been widely used in industrial production and human life [1]. However, the widespread burning of fossil fuels produces a large amount of carbon dioxide (CO_2_), which leads to a series of ecological issues, such as acid rain, rising sea levels, melting glaciers and so on. Obviously, it harms the living environment of human being in a deep extent [2]. In 2007, China surpassed the United States and became a country with the largest CO_2_ emission, which accounting for 35% of global CO_2_ emissions and growing at an average 3.2% p.a. [3]. Therefore, the issue of China’s carbon emissions has become the focus of global attention, how to stabilize economic growth while reducing CO_2_ emissions is a major challenge facing by the current society.

For reducing the CO_2_ emissions, many governments have taken a series of effective measures, such as adjusting industrial structure, improving energy efficiency, and reducing the operation of unnecessary energy intensive industries [4]. It should note that the most important step to promote energy conservation and emission reduction is to vigorously develop the high-tech industries. Because the high-tech industry, as a knowledge and technology intensive industry, has higher industrial growth rate with low energy consumption, which has an important significance for sustainable development. Especially, with the advent of the “third industrial revolution”, the high-tech industry innovation has gradually developed and reach its peak. Moreover, in order to promote the development of high-tech industries, for example, the German government has adopted the “High Technology Strategy 2020”, and launched “innovation partnership2020” and “Standard Innovation Program”; both the UK and Singapore have launched the “Innovation Voucher Scheme”(IVS); the Unite States has launched the “Star Wars Program”, formally known as the “Strategic Defense Initiative” (SDI); France and Japan have put forward industrial innovation plans to “create tomorrow’s products” and “Japan’s Digital Innovation Plan” (ICT) respectively. In addition, the Chinese government also attaches more and more attention on the development of high-tech industry, China classifies the high-tech industry as one of strategic emerging industries and vigorously promotes its industrial development [5]. In recent years, with the implementation of “National High-tech R&D (863) Program” and “China Torch Program”, China’s high–tech industries have made a substantial progress, and they are gradually replacing the traditional energy intensive industries. In 2015, investments in China’s high–tech industries reached 199.507 billion yuan, and its contribution rate for GDP reached 20.42% [6]. Among them, technological innovation is not only the core of the development of high-tech industries, but also the core driving force of China’s sustainable development [7]. The improvement of technological innovation level can promote the transformation and optimization of industrial structure, improve industrial production efficiency, reduce energy consumption and CO_2_ emission in the production process, so as to promote green economic growth [8]. Technological innovation mainly comes from two aspects: the improvement of overall R&D level and the improvement of efficiency. However, the improvement of the overall R&D level requires a lot of expenditure and the training of R&D personnel, which has a certain time lag. The improvement of technological innovation efficiency is the key way to optimize the allocation of innovation resources, reduce resource consumption and CO_2_ emission, and accelerate green transformation [9]. Therefore, this paper uses the innovation efficiency of high-tech industries to measure the industrial development level, and discusses its impact on CO_2_ emissions.

Although China has a vast territory, its resource distribution is unbalanced, and it will lead to different regional development, especially, CO_2_ emissions exist significant difference in different regions. Therefore, it is necessary to incorporate spatial factor to analyze the effect of innovation efficiency of high-tech industries on CO_2_ emissions. However, most of studies on energy conservation and emission reduction, in existing literature, are carried out under the framework of non-spatial econometrics, and the spatial effect of CO_2_ emissions is ignored [10, 11]. Therefore, based on the spatial perspectives, this paper analyzes the spillover effect of CO_2_ emissions in China, by constructing three spatial weight matrices and using spatial econometric model to empirical analyze the effect of innovation efficiency and decomposition efficiency of high-tech industry on CO_2_ emissions, and then it also further analyzes its direct, indirect and total effects to provide necessary theoretical support for reasonable formulating and effective implementing the policy of energy conservation and emission reduction in China.

## Literature review

As a global issue, carbon emissions have attracted unprecedented attention by scholars. In academia, the research on CO_2_ emissions is mainly carried out from two aspects. On the one hand, some scholars focus on discussing the sources of CO_2_ emissions from different industries, such as industry, agriculture, and transportation industry [12–16]. Wei et al. (2016) calculated the CO_2_ emissions of metal and non-metal mining, thermal power, construction and other industries in Beijing [17]. On the other hand, some papers analyze various factors that affecting CO_2_ emissions. Liu(2009) pointed out that the level of urbanization, energy consumption, population density and economic growth are major factors that affecting CO_2_ emissions [18]. Liddle(2014) found that urbanization level and energy structure are important factors that contribute to the increase of CO_2_ emissions [19]. And Huang and Jim Wu(2010) believed that the adjustment of industrial structure can reduce energy consumption, and then reduce CO_2_ emissions. It is obvious that the high-tech industry development has become the important driving force for optimizing and upgrading the regional industrial structure [20]. Chang(2015) also drew similar conclusions [21]. Bakhsh et al.(2017) believed that the market structure and openness to foreign country have certain effect on regional CO_2_ emissions [22].

As a technology intensive industry, the development of high-tech industry can effectively reduce energy consumption, which is of great significance to sustainable development [18]. Zhang and Liu(2015) studied the effect of ICT on CO_2_ emissions. The results indicated that the development of ICT industry in China contributes to reduce CO_2_ emissions and the ICT industry has a good development prospect, especially in eastern region [23]. Wang and Han (2016) explored the effects of information and communication technology industry (ICT) on CO_2_ emissions in China, and they found there is a long-term co-integration relationship exists between ICT industry and CO_2_ emissions in China [24]. Kong et al.(2016) found that high-tech industry can significantly promote the energy-saving technology of pulp and paper industry in 25 emerging countries, and thus reducing CO_2_ emissions [25]. Hasanbeigi et al.(2016) used system optimization method to measure the effect of high-tech industry on emission reduction of coal system, they found the high-tech industry reduces coal consumption by an average of 20.12 million tons each year [26]. Sgobbi et al.(2016) took Europe as the research sample, through the study of Marine high-tech industry, they pointed out that the improvement of Marine energy technology could reduce CO_2_ emissions [27]. Wu et al. (2016) found that accelerating the development of new energy industry is one of the best ways to reduce CO_2_ emissions at present [28]. By studying the cycle model of CO_2_ emissions from new energy vehicles, Liu et al.(2016) found that the effective use of new energy can significantly reduce the consumption of fossil fuels in China, thus significantly reduce CO_2_ emissions [29]. Weber and Cabras(2017) analyzed the effect of renewable energy high-tech industry on CO_2_ emissions in German. They found the rapid development of the renewable energy industry will significantly reduce the use of highly polluting coal, and thus contributing to reduce CO_2_ emissions [30]. Morrison and Golden(2017) put forward that the bioenergy high-tech industry contributes to reduce the coal consumption and CO_2_ emissions from coal-fired power plants in the United States [31]. Lee et al.(2017) studied the impact of high-tech industries on CO_2_ emissions in Asian countries, and they found that low-carbon, green and energy-saving technologies play an important role in reducing CO_2_ emissions [32]. McDowall et al.(2018) also reached similar conclusions [33].

As the core of “theoretical economic development”, put forward by Schumpeter(1934), innovation has become a research hotspot by many scholars [34]. The former studies have also confirmed that innovation plays a vital role in sustainable development [35–37]. Therefore, how to improve the innovation efficiency has become an important pathway to promote industry development. In general, there are two common methods to measure innovation efficiency, namely nonparametric Stochastic Frontier Analysis Model(SFA) and parametric Data Envelope Analysis Model (DEA) [38–42]. Samut and Cafrı(2016) measured the innovation efficiency of 29 OECD countries from 2000 to 2010 by using DEA, and they further analyzed the change of efficiency through the Malmquist and its decomposition index [43]. Ma(2015) took 29 large and medium–sized industrial enterprises in China as research objects, and applied two-stage DEA to analyze the innovation activities of high-tech industries in China [44]. Guan and Chen(2010) used relation network DEA to measure the innovation efficiency of high-tech industries in china, and constructed network framework system to study two sub-processes of research and development (R&D) and commercialization [45]. Chen et al.(2018) also divided the research of high-tech industries into two stages: R&D and commercialization, and evaluated the innovation efficiency by applying two-stage network DEA [46].

“The First Law of Geography”, put forward by Tobler(1970), suggests that there is a spatial correlation between things, and things with closer distance are more relevant than things with far distance [47]. Therefore, CO_2_ emissions in each region are not independent, but exist spatial correlation, in other words, CO_2_ emissions in one province are influenced not only by local factors, but also by neighboring provinces. Tian and Zhou(2019) figured out that understanding the spatial difference in CO_2_ emissions is a key element to reduce CO_2_ emissions, thus the spillover effect of CO_2_ emissions cannot be ignored [48]. Dong et al.(2016) used Analytical Network Process(ANP) method to explore the spatial distribution of renewable energy industry in China, and further analyzed its effect on energy and environment. The results indicated that the renewable energy is mainly concentrated in the west region, while the high-tech industries are more agglomerated in eastern coastal area [49]. Therefore, in order to promote R&D investment and technological innovation for high-tech industries and thus propel the development of new energy industry, it is crucial to explore the effect of high-tech industry on CO_2_ emissions from the perspective of space.

To sum up, although there have been many studies on the impacts of high-tech industries on CO_2_ emissions, there are sill some deficiencies. First of all, existing researches have not reached a consensus on the measurement method of innovation efficiency of high-tech industries, which results in certain deviation; Secondly, many scholars use traditional analytical methods to explore the effect of high-tech industries on CO_2_ emissions, however, it is obvious that the regression of econometric model is usually more reliable than the traditional non-econometric models [50]; Finally, existing papers regard the research samples as an independent unit, while ignore the regional differences within them. Therefore, based on the above analysis, the innovation points of this paper are as follows: firstly, this paper used DEA Global-Malmquist to systematically measure the innovation efficiency value of high-tech industries for each province in China from 2005 to 2016, and then it further analyzes its decomposition index; secondly, this paper measures the CO_2_ emissions from eight sources in 30 provinces of China, and by applying spatial geography factor to environmental economics, it discusses spatial spillover effect of CO_2_ emissions in China; thirdly, on the basis of considering the spatial characteristics of CO_2_ emissions, this paper constructs three spatial weight matrices, including geographic spatial weight matrix, economic geographic nested spatial weight matrix and innovation spatial weight matrix, and uses spatial econometric model to empirical study the effect of high-tech industry innovation efficiency and its decomposition index on CO_2_ emissions, then it further analyzes its direct, indirect and total effect; finally, this paper uses industrial solid waste to replace CO_2_ emissions for conducting regression again, in order to ensure the robustness of the model.

## Methodology

### Spatial weight matrix

The first step to examine the spatial correlation is the selection of spatial weight matrix. In general, spatial weight matrix is divided into three types: proximity spatial weight matrix, distance spatial weight matrix and economic distance spatial weight matrix [51, 52]. The three weight matrices are as follows:

$$W=\{\begin{array}{l} 1\;if\;i\;and\;j\;are\;adjacent\\ 0\;if\;i\;and\;j\;are\;not\;adjacent \end{array}$$ (1)

W={1dijifi≠j0ifi=j (2)

$$W=\{\begin{array}{l} 1/\left|{\bar{GDP}}_{i}-\bar{GDP_{j}}\right|\;if\;i\neq j\\ 0\;if\;i=j \end{array}$$ (3)

However, since the proximity spatial weight matrix cannot fully contain both geographic and economic development factor, and economic distance spatial weight matrix is easy to generate endogeneity problem, therefore, this paper uses the economic geographic nested spatial weight matrix instead of the economic distance spatial weight matrix. It not only considers the spatial influence between geographical distances, but also takes into account the regional spillover effect introduced by the economy, which can fully reflect the spatial correlation between regions.

$$W=\{\begin{array}{l} \varphi 1/d_{ij}+(1-\varphi )1/\left|{\bar{GDP}}_{i}-\bar{GDP_{j}}\right|\;if\;i\neq j\\ 0\;if\;i=j \end{array}$$ (4)

Where *d*_*ij*_ refers to the distance between provincial capitals i and j. ${\bar{GDP}}_{i}$ is the average of the GDP of province i during the research period. *φ* is between 0 and 1, which represents the proportion of the geographic distance spatial weight matrix. To simplify the analysis, we take it as 0.5.

Simultaneously, with regard to the particularity of high-tech industry itself, its industrial development depends on the innovation environment of the region to a certain extent. Thus, this paper incorporates regional innovation environment factors into the spatial weight matrix to form a new spatial weight matrix:

$$W=\{\begin{array}{l} 1/\left|\bar{R\&D_{i}}/{\bar{GDP}}_{i}-\bar{R\&D_{j}}/{\bar{GDP}}_{j}\right|\;if\;i\neq j\\ 0\;if\;i=j \end{array}$$ (5)

To sum up, this paper selects economic geographic nested spatial weight matrix (W_1_), geographic distance spatial weight matrix (W_2_) and innovative spatial weight matrix (W_3_).

### Spatial autocorrelation test

#### Global Moran’s Index

It is obvious that Moran’s Index (Moran’s I) is the most commonly used method to examine the spatial autocorrelation, the formula is shown as follows:

Moran'sIit=1∑i=1n∑j=1nwij×∑i=1n∑j=1nwij(xit−x¯)(xjt−x¯)∑i=1n(xit−x¯)/n (6)

Where $\bar{x}=\frac{1}{n}\sum_{i=1}^{n}x_{it}$, *x*_*i*_ represents the value of CO_2_ emissions for the i^th^ province at the t year. *W*_*ij*_ denotes the distance between i and j regions, which representing the (i, j) elements in the spatial weight matrix, $\sum_{i=1}^{n}\sum_{j=1}^{n}w_{ij}$ represents the sum of all elements of spatial weight. *MI*∈(−1,1), if MI > 0, it indicates that there is a positive spatial correlation, that is, high value and high value are clustered together (low value and low value are clustered together); conversely, if MI < 0, it means that there is a negative spatial correlation, which means high value and low value are adjacent; if MI = 0 indicates that there is no spatial correlation, that is, the distribution of high value and low value are completely random.

#### Local Moran’s Index

In order to further analysis the spatial correlation of CO_2_ emissions, this paper introduced Local Moran’s I to further conduct spatial test. The formula is shown as follows:

$$Moran's\;I_{it}=\frac{\left(x_{it}-\bar{x})\sum_{j=1}^{n}W_{ij}(x_{jt}-\bar{x}\right)}{\sum_{i=1}^{n}{\left(x_{i}-\bar{x}\right)}^{2}/n}$$ (7)

Where, the meaning of all symbols in Eq (7) is consistent with Eq (6).

### Spatial panel Durbin model (SDM)

This paper constructs Spatial Durbin model to empirical analyze the effect of innovation efficiency of high-tech industries on CO_2_ emissions, the model is shown as follows:

y=λWy+Xβ+μ (8)

The generation process of disturbance term *μ* is:

$$\mu =\rho M\mu +\varepsilon ,\varepsilon ∼N(0,\sigma^{2}I_{n})$$ (9)

Where, *W* and *M* are the spatial weight matrix of explained variable *y* and disturbance term *μ* respectively. When *λ* = 0, the model is shown as follows:

$$CR_{it}=\rho W_{ij}CR_{it}+\beta_{1}X_{it}+\beta_{2}X_{it}+\mu_{it}+\gamma_{it}+\varepsilon_{it}$$ (10)

$$\mu =\rho M\mu_{it}+\varepsilon_{it},\varepsilon ∼N(0,\sigma^{2}I_{n})$$ (11)

Where, *CR*_*it*_ is the explained variable, representing the CO_2_ emissions of the i^th^ province (autonomous region, municipality directly under the central government) in period t; *X*_*n*_ is the explanatory variable; *W*_*ij*_ is the proximity spatial weight matrix; *W*_*ij*_*X*_*ij*_ is the spatial lag term; *μ*_2_*W*_*ij*_*X*_*ij*_ denotes the effect of neighboring provinces on the local region; *ρ* is the coefficient of the spatial lag term of the interpreted variable; *β*_1_ is the coefficient of the explanatory variable, and *β*_2_ is the coefficient to explain the lag term of variable space; *μ*_*it*_ and *λ*_*it*_ represent spatial effect and time effect respectively, and *ε*_*it*_ denotes interference item.

## Data

### Dependent variable

CO_2_ emissions: this paper uses the standard coal method for reference, provided by “2006 IPCC guidelines for National Greenhouse Gas Inventory” (Table 1), and measures CO_2_ emissions of 30 provinces from 2006 to 2016, based on 8 types of energy consumption (coal, coke, crude oil, gasoline, kerosene, diesel, natural gas and fuel oil), the formula is shown as follows:

$$CO_{2ti}=\sum_{j=1}^{8}CO_{2tij}=\sum_{i=1}^{8}E_{tij}*NCV_{j}*CEF_{j}*COF*44/12$$ (12)

Where, t represents the year, i denotes province, j represents the energy; *CO*_2*ti*_ refers to CO_2_ emissions of the i^th^ province in t year, *E*_*tij*_ denotes the consumption of j energy for i province in year t, *NCV*_*j*_ represents the net calorific value of the j^th^ energy, *CEF*_*j*_ is carbon emission factor for the j^th^ energy, *COF* is the carbon oxidation factor; 44/12 represents the carbon is oxidized to CO_2_, the molecular weight change from 12 to 44, therefore, it should make a corresponding transformation when calculating CO_2_ emissions.

**Table 1:** Carbon emission coefficients of various energy.

Energy Category	Lower calorific value *F*_*j*_ (kJ/kg)	Carbon Emission Factor *C*_*j*_ (tC/TJ)	Carbon Oxidation Factor	Carbon Emission Coefficient (gc/Kgce)
**Coal**	20908	26.4	0.94	1.9003
**Coke**	28435	29.5	0.93	2.8604
**Crude Oil**	41816	20.1	0.98	3.0202
**Fuel Oil**	41816	21.1	0.98	3.1705
**Gasoline**	43070	18.9	0.98	2.9251
**Kerosene**	43070	19.5	0.98	3.0179
**Diesel**	42652	20.2	0.98	3.0959
**Natural Gas**	38931	15.3	0.99	2.1622

Data source: IPCC (2006) and Energy research institute of National Development and Reform Commission (2007).

### Core independent variable

High-tech industry innovation efficiency (TFP): Malmquist index is an application method, proposed by Malmquist(1953) [53], when studying the consumption changes in the same period. Caves et al.(1928) used Malmquist index to the measurement of productivity for the first time [54]. Then, many scholars combined this index with the Data Envelopment Analysis Method (DEA), proposed by Charens et al.(1978), in order to widely use the index for measuring changes in productivity from period t to period (t+1) [38, 55–58]. The specific methods are as follows:

By setting the t period as the based period, the input productivity index can be defined as:

$$M^{t}=d^{t+1}(x^{t+1},y^{t+1})/d^{t}(x^{t},y^{t})$$ (13)

Then, the productivity index based on (t+1) can be defined as:

$$M^{t+1}=d^{t+1}(x^{t+1},y^{t+1})/d^{t+1}(x^{t},y^{t})$$ (14)

Fare et al. constructed the Malmquist index production efficiency model from period t to t+1 and further decomposed the index [59]. Therefore, the Malmquist index model from the input angle is as follows

M(xt+1,yt+1,xt+1,yt+1)={dt+1(xt+1,yt+1)dt+1(xt,yt)dt(xt+1,yt+1)dt(xt,yt)}12=dt+1(xt+1,yt+1)dt(xt,yt)[dt(xt+1,yt+1)dt+1(xt+1,yt+1)dt(xt,yt)dt+1(xt,yt)}12=EC*TC (15)

Where, d^*t*^(*x*^*t*^,*y*^*t*^) and d^*t*^(*x*^*t*+1^,*y*^*t*+1^) represents the technical efficiency level at period t and period (t+1) when taking the technology of period t as a benchmark. d^*t*+1^(*x*^1^,*y*^*t*^) and d^*t*+1^(*x*^*t*+1^,*y*^*t*+1^) is the technical efficiency level at period t and period (t+1) when taking the technology of period (t+1) as a benchmark. If *M*>1, it shows the productivity from period t to period t+1 will increase; otherwise, it will decrease. Furthermore, the index can be further decomposed into EC and TC, EC represents the change in technical efficiency, it is used to measure the catch-up angle from period t to period t+1 for each observed object to the best practice boundary with constant return to scale, and TC represents the technical progress efficiency, and it is used to measure the movement of technology boundary from t period to t+1 period with constant return to scale.

In general, the innovation input index is measured from two aspects, that is, the input of manpower and capital. For the manpower input, this paper selects full time equivalent of R&D personnel in high-tech industries to represent the manpower input index. For the capital input, many scholars use internal expenditure on R&D to express. However, the internal funds expenditure on R&D is a flow indicator, it will not only affect the current innovation activity but also affect innovation output in the later period due to the cumulative effect. Therefore, this paper used the perpetual inventory method to calculate capital stock of R&D [60], and the specific formula is shown in Eq (16):

$$K_{t}=(1-\tau )K_{t-1}+I_{t}$$ (16)

Where, *K*_*t*_ represents the capital stock in period t; *K*_*t*−1_ denotes the actual capital stock in period t-1; *I*_*t*_ represents the real internal expenditure on R&D in period t; *τ* represents the depreciation rate, and this paper used weighted average of fixed asset price index and consumption price index to carry out calculation. In order to calculate the capital stock *K*_*t*_ of each period, the first step is to determine the capital stock in the base period, *K*_0_, assuming the growth rate of R&D capital stock is equal to the growth rate of actual R&D internal expenditure, and the formula is shown in Eq (17):

$$K_{0}=E_{0}/(g+\tau )$$ (17)

Where, *K*_0_ is the capital stock in the base period; E_0_ denotes the actual R&D internal expenditure; g is the average value of the growth rate of actual R&D internal expenditure in each period.

In addition, the selection of innovation output indicator is mainly from two aspects: firstly, it should select the output indicator from the aspect of the technology research and development level, due to the number of patent applications could more clearly reflect the technological innovation capability of the industries, and the lag period does not have significant impact on it, this paper selects the number of patent applications as one of the output indicators. Secondly, it should select the output indicator from the aspect of the economic conversion capacity, obviously, the way for high-tech industries to gain economic benefits is to convert the technology research and development achievements into new product output. Therefore, this paper selects the sales revenue of new product in high-tech industries as the second output indicator.

### Control variables

In addition to the effect of innovation efficiency in high-tech industries on CO_2_ emissions, it also needs to consider the effect of other factors on CO_2_ emissions. Therefore, in order to further explore high-tech industries (TFP) and its effect of the decomposed index, change in technical efficiency (EC) and efficiency of technical progress (TC), on CO_2_ emissions, methods are shown in Table 2.

**Table 2:** Calculation method.

Variable Name	Variable Symbol	Calculation Method	Unit
**CO**_**2**_ **emissions**	CO_2_	Obtained from the above calculation Eq (12)	10,000 tons
**Innovation efficiency**	TFP	Obtained from the above calculation	
	TFP^2^		
	TFP^3^		
**Changes in Technical Efficiency**	EC	Obtained from the decomposition of TFP	
	EC^2^		
	EC^3^		
**Technical progress Efficiency**	TC	Obtained from the decomposition of TFP	
	TC^2^		
	TC^3^		
**Open degree**	FDI	Foreign Direct investment/GDP	%
**Energy consumption**	EI	Total energy consumption	10,000 tons
**Market environment**	ME	Technology market turnover	10,000 yuan
**Industrial structure**	INS	Added value of the secondary and tertiary industries/GDP	%
**Urbanization level**	URB	Year-end urban population / Year-end total population	%

### Data source

This paper takes 30 provinces in mainland China (excluding Tibet with incomplete data), from 2005 to 2016, as the research object, the data are all from the“China Statistical Yearbook”, “China Statistical Yearbook on High Technology Industry”and “China Statistical Yearbook on Environment”. It uses the distance between provincial capitals to represent the distance between each province, and the longitude and latitude coordinates of cities are obtained from 1: 4000000 topographic database of the National Fundamental Geographic Information System (NFGIS). Moreover, by choosing 2000 as the based year, all monetary indicators are deflated by using the fixed asset investment price index and consumption price index. In addition, for eliminating the effect of heteroscedasticity, this paper takes a logarithm treatment for ME. The specific data description is shown in Table 3.

**Table 3:** Summary statistics.

Var	Obs	Mean	Std	Min	Max
**CO**_**2**_	330	0.359	0.255	0.020	1.406
**TFP**	330	1.198	0.634	0.187	5.635
**EC**	330	1.155	0.515	0.222	3.649
**TC**	330	1.076	0.439	0.289	5.635
**FDI**	330	0.029	0.023	0.001	0.122
**EI**	330	11100.97	7746.70	520	38899
**INS**	330	0.890	0.056	0.673	0.996
**lnME**	33	3.781	1.772	-0.626	8.279
**URB**	330	0.530	0.140	0.275	0.896

## Results

### Empirical results analysis of CO_2_ emissions

This paper calculates CO_2_ emissions of 30 provinces in China (excluding Tibet, Hong Kong,Taiwan and Macao) based on the above carbon emission calculation Eq (12). However, due to space limitations, this paper only lists the CO_2_ emissions in 2006, 2008, 2010, 2012, 2014, 2016 and average CO_2_ emissions from 2006 to 2016. As shown in Table 4, on average, from 2006 to 2016, the average CO_2_ emissions of 30 provinces in China is 0.360 billion tons. Shandong has the highest CO_2_ emissions, which is 1.099 billion tons, obviously, it is the only province in China with more than 1,000,000,000 tons of CO_2_ emissions. Next are Hebei, Shanxi and Jiangsu, which are 0.809, 0.711, 0.691 billion tons respectively. Qinghai (0.042 billion tons) and Hainan (0.050 billion tons) have the lowest CO_2_ emissions, less than 0.05 billion tons. In addition, Hebei, Shanxi, Inner Mongolia, Liaoning, Zhejiang, Shandong, Henan and Guangdong are all above the national average level and most of them are located in eastern region. From the perspective of time, the average CO_2_ emissions of 30 provinces in china increase every year from 2006 to 2016. However, it should note that the CO_2_ emissions of other regions start to decline, excepting Anhui, Fujian, Jiangxi, Shandong, Hainan, Sichuan, Shanxi, Qinghai, Ningxia, and Xinjiang, in particular, each province in eastern region, there is a significant decrease in CO_2_ emissions.

**Table 4:** CO_2_ emissions (billion tons).

	2006	2008	2010	2012	2014	2016	Main
**Beijing**	0.115	0.122	0.124	0.114	0.101	0.082	0.110
**Tianjin**	0.131	0.137	0.180	0.198	0.194	0.174	0.171
**Hebei**	0.619	0.703	0.805	0.921	0.874	0.864	0.809
**Shaanxi**	0.621	0.630	0.665	0.765	0.800	0.766	0.711
**Neimeng**	0.363	0.499	0.597	0.778	0.777	0.783	0.640
**Liaoning**	0.533	0.591	0.670	0.734	0.697	0.687	0.654
**Jilin**	0.200	0.217	0.247	0.280	0.266	0.243	0.244
**Heiljiang**	0.261	0.297	0.338	0.379	0.359	0.365	0.333
**Shanghai**	0.225	0.241	0.261	0.261	0.246	0.254	0.250
**Jiangsu**	0.512	0.564	0.659	0.771	0.779	0.833	0.691
**Zhejiang**	0.336	0.382	0.424	0.433	0.424	0.424	0.409
**Anhui**	0.211	0.268	0.312	0.348	0.386	0.385	0.322
**Fujian**	0.147	0.172	0.222	0.250	0.276	0.249	0.222
**Jiangxi**	0.128	0.141	0.172	0.190	0.206	0.217	0.177
**Shandong**	0.782	0.938	1.078	1.192	1.236	1.406	1.099
**Henan**	0.476	0.543	0.600	0.614	0.609	0.602	0.582
**Hubei**	0.264	0.291	0.358	0.407	0.351	0.348	0.339
**Hunan**	0.241	0.261	0.291	0.319	0.297	0.315	0.292
**Guangdong**	0.428	0.472	0.565	0.597	0.589	0.604	0.545
**Guangxi**	0.113	0.128	0.172	0.232	0.227	0.227	0.184
**Hainan**	0.020	0.041	0.048	0.057	0.059	0.064	0.050
**Chongqing**	0.091	0.123	0.146	0.163	0.148	0.146	0.137
**Sichuan**	0.221	0.277	0.312	0.334	0.352	0.313	0.305
**Guizhou**	0.211	0.210	0.232	0.280	0.280	0.294	0.253
**Yunnan**	0.193	0.208	0.239	0.256	0.226	0.200	0.223
**Sxi**	0.209	0.257	0.332	0.423	0.472	0.477	0.360
**Gansu**	0.136	0.154	0.169	0.201	0.208	0.195	0.179
**Qinghai**	0.025	0.036	0.037	0.050	0.052	0.055	0.042
**Ningxia**	0.079	0.098	0.127	0.182	0.198	0.203	0.150
**Xinjiang**	0.163	0.198	0.261	0.358	0.446	0.490	0.318
**Main**	0.268	0.307	0.355	0.403	0.404	0.409	0.360

**Fig 1:**
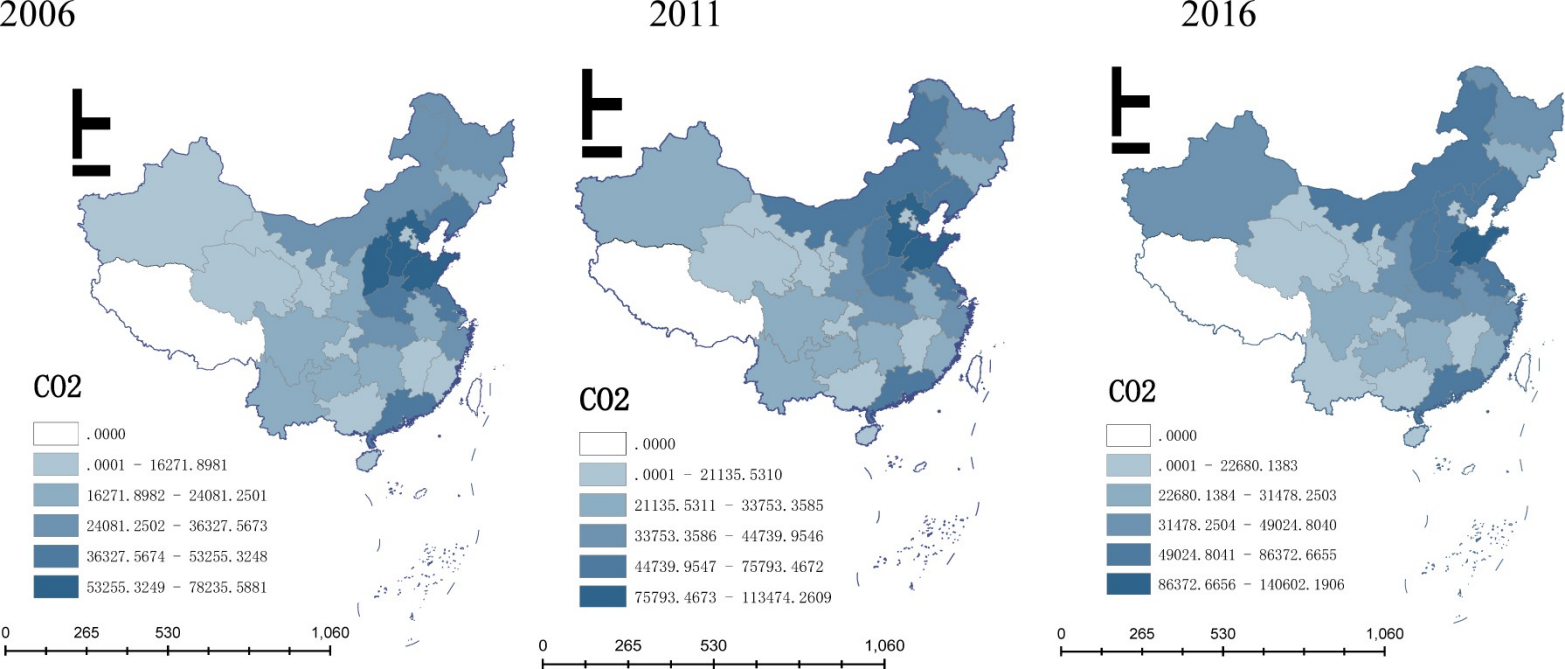
Spatial distribution diagram of CO_2_ emissions in China.

In order to further analyze the spatial distribution situation of CO_2_ emissions, this paper uses ArcMap10.7 to draw the spatial distribution diagram of CO_2_ emissions in 2006, 2011 and 2016 (as shown in Fig 1). From the perspective of space, in 2006–2016, CO_2_ emissions present a decreasing trend from eastern coastal region to middle and western region. The region with high CO_2_ emissions mainly concentrates on eastern coastal area (Shandong, Hebei, Liaoning, Jiangsu etc.)and some inland provinces, such as Inner Mongolia, Henan, and Guangdong. It is obvious that the Bohai Rim region and Yangtze River delta are major regions with high CO_2_ emissions, and it has formed a continuous belt of high emissions in general, that is “Inner Mongolia–Hebei–Liaoning–Jiangsu–Zhejiang”. This may be due to the secondary industry is well-developed in the eastern region, especially, some provinces, such as Hebei and Shandong, have large share of heavy industry, and it will make these provinces lead the country in CO_2_ emissions.

### Empirical results analysis of innovation efficiency in high-tech industry

As shown in Table 5, from 2005 to 2016, the mean value of innovation efficiency in high-tech industries (TFP) for 30 provinces in China is 1.1977, which indicates the overall innovation efficiency of high-tech industries in China presents an upward trend with an average annual increase of 19.8%. The technical efficiency (EC) and technical progress (TC) also show an upward trend in general, with average annual increase of 15.5% and 7.6% respectively. From the time view, in 2006–2007, the average innovation efficiency in high-tech industry rose to 1.363, and it had reached its peak value in the recent decade. Although the growth rate of TC decreases, the significant increase in EC promotes the growth of TFP. After 2007, the growth rate of TFP started to decrease, it may be affected by the drop of TC, however, the overall fluctuation is not significant, and TFP remains steady growth until 2013. In 2013–2014, the growth rate of TFP experienced a significant decrease, only 1.093, although the TC increased by 4.3%, it still cannot make up for the significant decline in EC. However, in 2014–2015, due to the TC further increased to 1.254, the TFP rebounded to 1.325 after experiencing a short decline. In 2015–2016, EC increased to 1.340, however the TFP only increased by 21.9%, due to the decline of TC. In addition, except for 2005–2006 and 2011–2012, the EC is greater than 1, which means the technical efficiency of high-tech industries declined in 2005–2006 and 2011–2012. This may be due to the mismatch of factors, such as capital and manpower, during these periods. For the technical progress index, the TC of some years is less than 1, this may due to the high-tech industries in China lack R&D motivation, or excessive investments cause waste of resources, thus reducing the production technology innovation level.

**Table 5:** Innovation efficiency and its decomposition index.

Year	TFP	EC	TC	Year	TFP	EC	TC
**2005–2006**	1.119	0.825	1.407	2011–2012	1.120	0.927	1.216
**2006–2007**	1.363	1.165	1.183	2012–2013	1.218	1.293	0.976
**2007–2008**	1.170	1.239	0.999	2013–2014	1.093	1.044	1.043
**2008–2009**	1.141	1.306	0.893	2014–2015	1.325	1.056	1.254
**2009–2010**	1.117	1.233	0.926	2015–2016	1.219	1.340	0.957
**2010–2011**	1.289	1.282	0.986	Main	1.198	1.155	1.076

**Fig 2:**
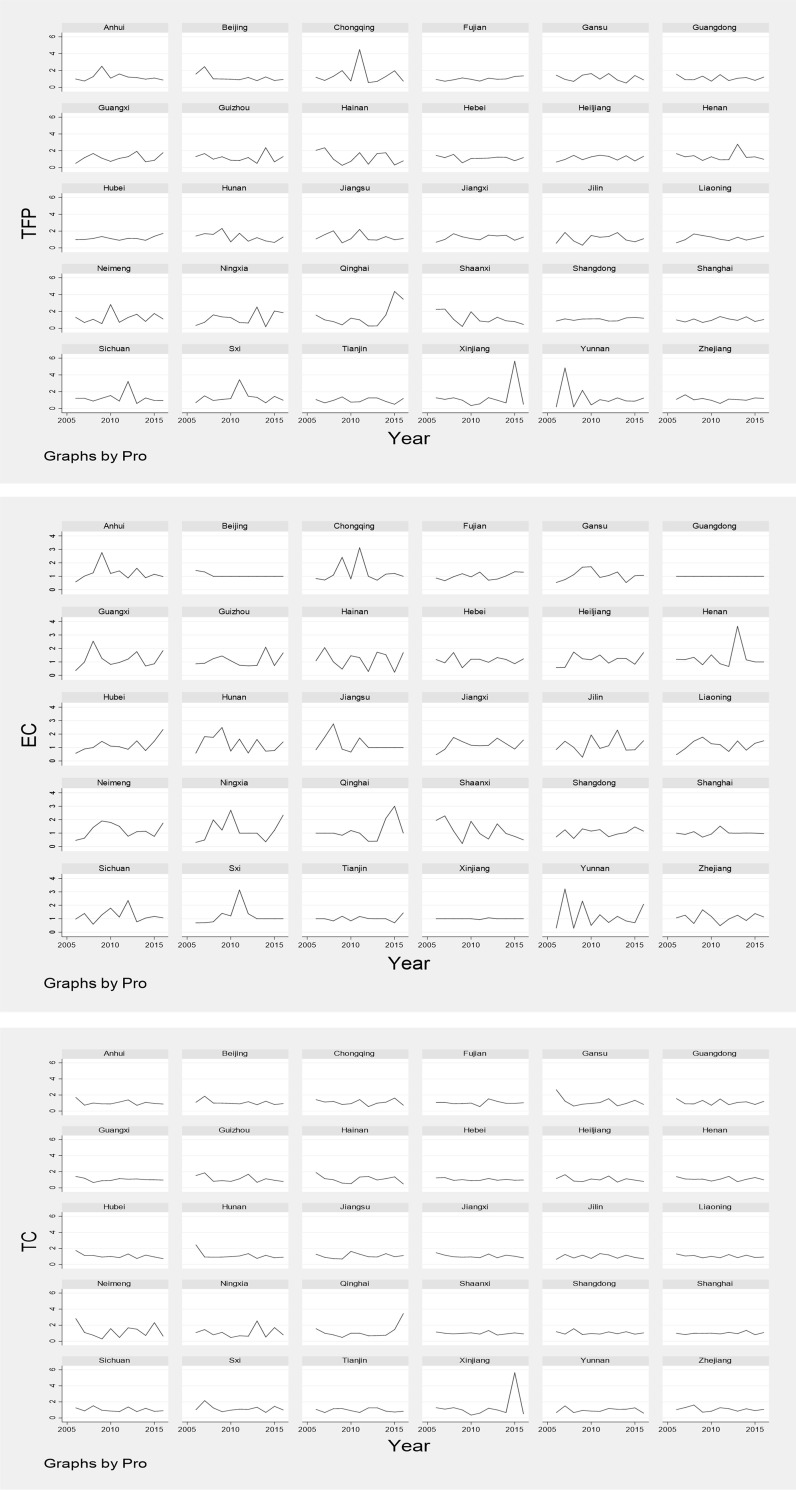
The variation trend of TFP and its decomposition index.

As shown in Fig 2, in 2005–2016, in addition to Chongqing, Qinghai, Xinjiang and Yunnan, the TFP in other provinces was relatively stable. The change of TFP in high -tech industries of Chongqing, Qinghai and Yunnan mainly depend on the substantial increase of EC, while Xinjiang is more affected by TC. This may be because, in recent years, the government has vigorously developed the western region and provided more policy support to western region. Meanwhile, it is obvious that “One belt and one road” also brings many new opportunities for the development of western region, promotes the improvement of its technical efficiency level. Furthermore, the TFP of high-tech industries in eastern region, such as Fujian, Shandong and Shanghai etc., maintains a steady growth. In addition, the variation trend of TC and TFP in each province is basically same, and the change trajectory is also similar, which indicates the TC is the major factor to affect the change of TFP in high-tech industries for each province in China.

**Fig 3:**
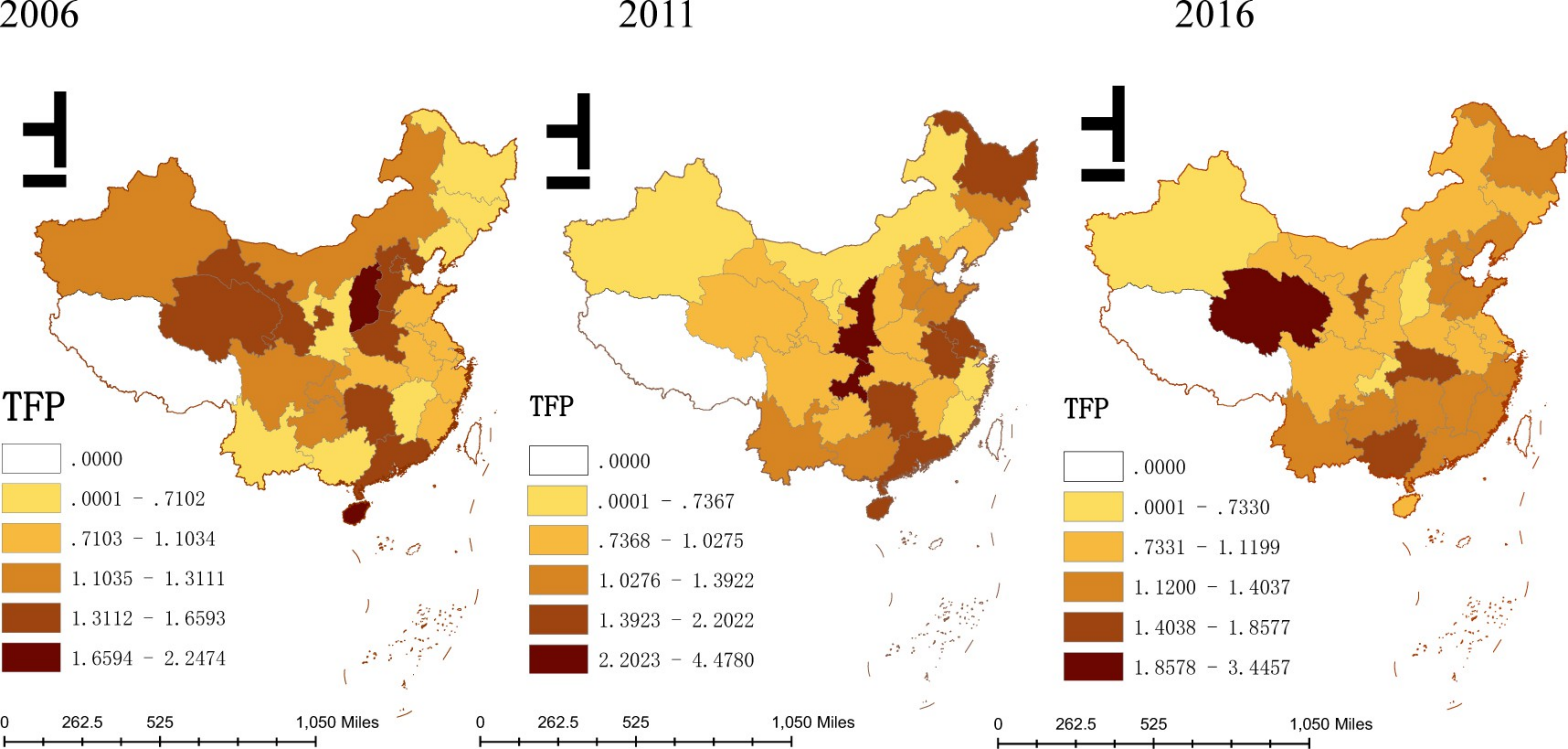
Spatial distribution of TFP.

As shown in Fig 3, the innovation efficiency of high-tech industries in each province exist significant spatial differences. In 2006, except for Beijing and Hebei, other provinces in eastern region had a relative low innovation efficiency of high-tech industries. On the one hand, it may due to the innovation of high-tech industries itself exist a lag. On the other hand, it may be because the eastern region bears excessive homogeneous R&D investment, which will lead to waste of resources and low innovation efficiency. From 2011 to 2016, the achievements of R&D investment in high-tech industries in all regions is increasing prominent. With the exception of some regions, innovation efficiency of high-tech industries in each province has been improved, especially the province in eastern coastal area, such as Liaoning, Zhejiang, Shandong and Fujian etc.

### Baseline results

The baseline results, including RE、FE and ME are shown in the Table 6. The results show that the regression results of TFP in FE, RE and ME model all show an inverted “N” shape, which means the effect of TFP on CO_2_ emissions presents a characteristic of “lower–higher–lower”, however such effect is not significant. In terms of control variables, the regression coefficient of URB are all significantly positive at 1% significance level, that is, for every 1% increase in urbanization level, CO_2_ emissions will increase by about 3%. Obviously, the impact of urbanization level on CO_2_ emissions comes from two aspects. On the one hand, the improvement of urbanization level will make the population gather in the urban center, which will improve the service efficiency of urban infrastructure, public goods, urban landscaping and so on. On the other hand, the increase of urban population also intensifies the usage of urban buildings, automobile and so on, which may damage the environment. Therefore, the combined effect of two aspects makes the impact of urbanization level on CO_2_ emission become more complicated. As the regression coefficients of FDI are not statistically significant in the most models, the influence of the level of openness on CO_2_ emissions is difficult to be reflected in the baseline regression model. In the most models, the EN、lnME variables do not obtain consistent and significant regression results.

**Table 6:** Baseline results.

	(1)	(2)	(3)	(4)	(5)	(6)	(7)	(8)	(9)
	RE			FE			ME		
**TFP**	0.025**	-0.038	-0.088	0.024**	-0.036	-0.910	0.025**	-0.038	-0.088
	(2.28)	(-1.13)	(-1.36)	(2.31)	(-1.10)	(-1.37)	(2.28)	(-1.13)	(-1.36)
**TFP** ^**2**^		0.015**	0.042		0.014**	0.044		0.015**	0.422
		(2.55)	(1.46)		(2.56)	(1.49)		(2.55)	(1.46)
**TFP** ^**3**^			-0.004			-0.004			-0.004
			(-0.99)			(-1.06)			(-0.99)
**FDI**	-0.679	-0.644	-0.692	-0.297	-0.275	-0.275	-0.679	-0.644	-0.692
	(-0.60)	(-0.59)	(-0.63)	(-0.27)	(-0.26)	(-0.26)	(-0.60)	(0.59)	(-0.63)
**LnME**	0.001	0.003	0.005	-0.014	-0.010	-0.011	0.001	0.003	0.005
	(0.01)	(0.07)	(0.11)	(-0.31)	(-0.25)	(-0.25)	(0.00)	(0.07)	(0.11)
**EN**	0.156	0.146	0.130	0.256	0.244	0.239	0.156	0.146	0.129
	(0.92)	(0.88)	(0.79)	(1.48)	(1.44)	(1.43)	(0.92)	(0.88)	(0.79)
**INS**	2.230	2.197	2.238	1.962	1.936	1.943	2.229	2.197	2.238
	(1.55)	(1.55)	(1.57)	(1.44)	(1.44)	(1.44)	(1.55)	(1.55)	(1.57)
**URB**	3.042***	2.990***	2.927***	3.518***	3.454***	3.454***	3.042***	2.990***	2.927***
	(3.02)	(3.17)	(3.09)	(3.42)	(1.44)	(1.43)	(0.92)	(3.17)	(3.06)
**R** ^**2**^	0.621	0.626	0.627	0.622	0.628	0.629	0.621	0.626	0.627
**obs**	330	330	330	330	330	330	330	330	330

Note: Figures in parentheses are Z-value.

***, **and * indicate the significance at 1%,5% and 10% levels respectively.

### Spatial autocorrelation analysis

**Fig 4:**
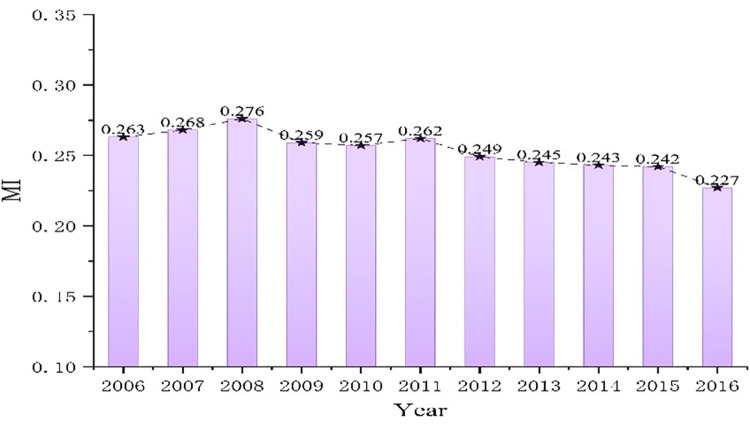
Changing trend of Moran’s I.

Table 7 shows the Moran’s I of CO_2_ emissions in China from 2006 to 2016, and its changing trend is plotted in Fig 4. In 2006–2016, Moran’s I is significant positive at 1% significance level, that is, it indicates the CO_2_ emissions of 30 provinces in China exist significant positive spatial correlation, which means the CO_2_ emissions of each province are not randomly distributed, while it will be affected by the CO_2_ emissions level of neighboring provinces, which presents a geographically agglomerated phenomenon. From the time quantum, the Moran’s I of CO_2_ emissions in provincial region shows a downward trend, especially after 2008, the downward trend is significant, and it indicates that the interaction effect of CO_2_ emissions between provinces decreases.

**Table 7:** Moran’s Index of CO_2_ emission.

Year	MI	Z	Year	MI	Z
**2006**	0.263***	2.469	2012	0.249***	2.370
**2007**	0.268***	2.519	2013	0.245***	2.330
**2008**	0.276***	2.599	2014	0.240***	2.307
**2009**	0.259***	2.456	2015	0.242***	2.354
**2010**	0.257***	2.440	2016	0.227***	2.251
**2011**	0.262***	2.465			

Note:

***, **and * indicate the significance at 1%, 5% and 10% levels respectively.

**Fig 5:**
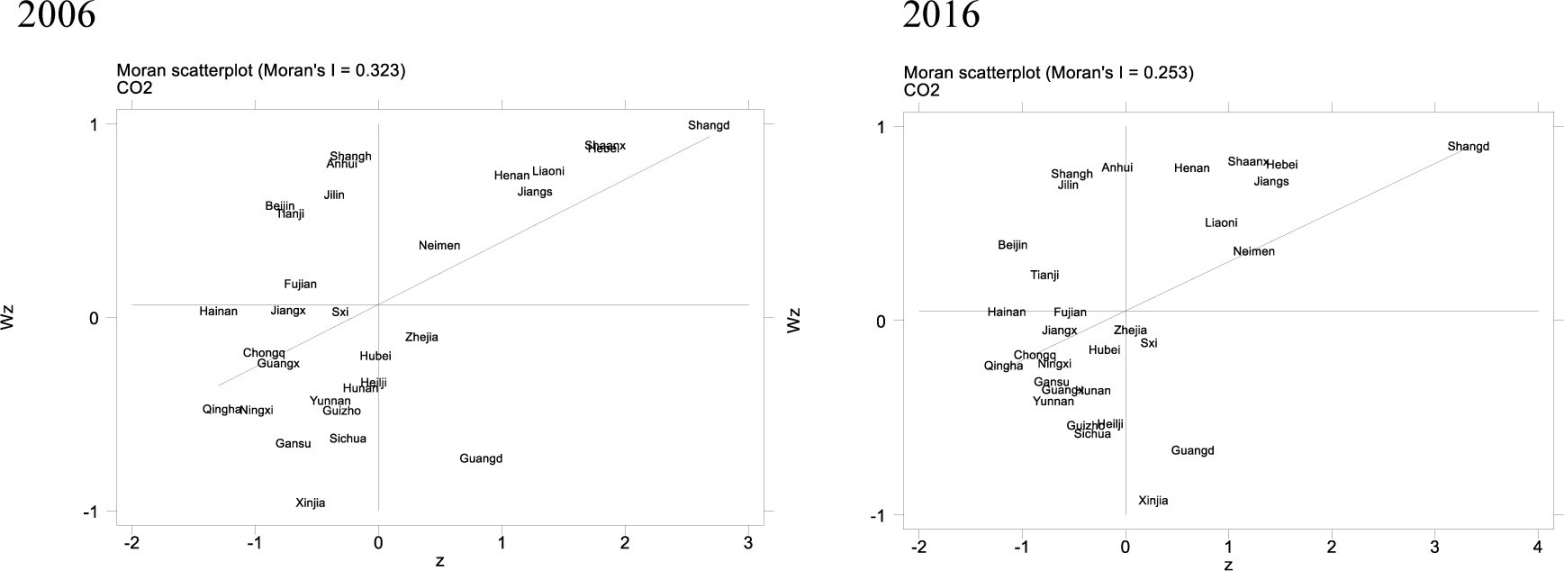
Moran’s I scatter diagram of CO_2_ emissions in provinces of China.

This paper uses scatter diagram to report the result of Local Moran’s I. the horizontal axis represents the current value of sample variables, and the vertical axis represents the spatial lag term. In the diagram, the spatial correlation between the sample region and the adjacent region is divided into four parts: “high-high” (HH), “high -low”(HL), “low-low” (LL), and "low-high" (LH). It should note that "high-high" (HH) and "low-low" (LL) indicate that if the sample area is high value (low), then the adjacent area is also high value (low), which means the study sample exists a positive spatial correlation. Conversely, the study sample exist a negative spatial correlation. Due to the large calculation quantity of local Moran’s I, this paper will not list the detailed calculation results, and it only reports the Moran’s I scatter diagram in 2006 and 2016 (Fig 5). By comparing the Moran’s I scatter diagram of 2006 and 2016, this paper found that the quadrants and positions for each province does not change significantly when the timing sequence changes, and it indicates that the CO_2_ emission level in China has a relatively stable spatial correlation. For example, Beijing, Tianjin, Jilin, Anhui and Shanghai are always located in the first quadrant, and the CO_2_ emissions show a spatial agglomeration characteristic of “high-high” (HH); While Hainan, Jiangxi, Hubei, Chongqing, Qinghai, Gansu, Hunan, Yunnan, Guangxi, Guizhou and Heilongjiang locate in the third quadrant, and the CO_2_ emissions show a spatial agglomeration characteristic of “low-low” (LL); For the other provinces, located in the second and fourth quadrant, they shows a agglomeration characteristic of “high -low”(HL) and "low-high" (LH).

### Estimation results of the spatial panel Durbin model

In order to determine whether it is appropriate to construct a spatial econometric model in our study, it needs to conduct (Robust) LM test at first, including (Robust) LM lag test and (Robust) LM error test. The results show that LM lag and error test are significantly positive at 1% statistical level, indicating that the model residuals have spatial dependent. Next, if the spatial correlation exists in spatial panel model, the paper should construct a general Spatial panel Durbin model (SDM) to confirm whether the SDM would be transformed into the spatial lag model (SLM) or the spatial error model (SEM) through the LR and Wald test [61]. According to Table 8, it finds that the results of LR and Wald test are all significant at the 1% statistical level, which show that the SDM model cannot be simplified into SEM or SLM. Moreover, the Hausman results show that the fixed effect should be applied. Therefore, based on the test results in Table 8, we select the SDM under the fixed effect to empirically analyze the impact of innovation efficiency of high-tech industry on the CO_2_ emission.

**Table 8:** Spatial models specification results.

Test	Statistics	Test	Statistics
**LM (lag)test**	9.460***	LR test spatial lag	41.38***
**Robust LM (lag)test**	1.744	LR test spatial error	41.17***
**LM (error) test**	9.826***	Wald test spatial lag	43.94***
**Robust LM (error) test**	2.113	Wald test spatial error	44.91***
**Hausman test**	61.23***		

Note:

***, **and * indicate the significance at 1%, 5% and 10% levels respectively.

Tables 9–11 respectively report the regression results of SDM model under three spatial weight matrices when the major explanatory variables are TFP, EC and TC, and by adding the quadratic term and the cubic term for TFP, EC and TC, it finds that the results of adding the cubic term are more accurate. By comparing the Tables 9–11, under the W_1_ and W_2_, the spatial lag term coefficients of CO_2_ emissions are significantly negative at 1% significance level, which indicates that the CO_2_ emissions of China’s provincial regions have the characteristics of spatial agglomeration, that is, CO_2_ emissions from adjacent regions have impact on the local region. For example, under W_1_, CO_2_ emissions from adjacent regions increases by 1%, the local region will reduce emissions by about 50%. However, under W_3_, such effect is not significant. From the perspective of space, the effect of innovation efficient in high-tech industries is not a simple linear relationship, according to the column (1), (4) and (7) of Table 9, the coefficients of TFP are 0.022, 0.029 and 0.026 respectively, and they all pass the significance test at 5% significance level. With the addition of quadratic term, the effect of TFP on CO_2_ emissions is no longer a simple linear relationship, but shows a “U-shaped” characteristic. By considering the particularity of the high-tech industry, the transformation of its innovation achievements may exist a lag, therefore, it is necessary to add the cubic term of TFP to discuss the effect of innovation efficiency of high-tech industries on CO_2_ emissions. From the results, the coefficients of the first and the cubic term of TFP are negative, the coefficients of the quadratic term are positive, which indicates CO2 emission presents a trend of inverted N shape curve with the growth of TFP. According to the column (3) of Table 9, in the early stage, if the TFP of high-tech industries increases by 1%, the CO_2_ emissions in local region will be reduced by 0.1%, with the further study on innovation, innovation inputs will increase significantly, it is obvious that CO_2_ emissions will also increase with the improvement of TFP, that is, if TFP increases by 1%, CO2 emissions will increase by about 0.05%; in the later stage, the innovation achievements of the previous stage have taken initial shape, the inhibiting effect on CO_2_ emissions starts to emerge, however, it still have a long time to reach the goal at present. At the same time, by changing the explanatory variables to EC and TC, as shown in Tables 10 and 11, it can be found that the coefficients of the first and cubic term of EC and TC, in most models, are negative, while the coefficients of the quadratic term is positive, this is consistent with the regression results of TFP.

**Table 9:** Estimation results of SDM.

	(1)	(2)	(3)	(4)	(5)	(6)	(7)	(8)	(9)
	W_1_			W_2_			W_3_		
**W-lnCO**_**2**_	-0.530***	-0.519***	-0.556***	0.793***	-0.776***	-0.789***	0.060	0.042	0.038
	(-2.74)	(-2.69)	(-2.87)	(-3.21)	(-3.16)	(-3.21)	(0.47)	(0.32)	(0.30)
**TFP**	0.022**	-0.028	-0.101*	0.029***	-0.023	-0.081	0.026***	-0.037	-0.082
	(2.50)	(-1.17)	(-1.65)	(3.52)	(-1.03)	(-1.46)	(2.78)	(-1.50)	(-1.39)
**TFP** ^**2**^		0.012**	0.051*		0.013**	0.044		0.015***	0.040
		(2.25)	(1.65)		(2.46)	(1.56)		(2.70)	(1.33)
**TFP** ^**3**^			-0.005			-0.004			-0.003
			(-1.28)			(-1.14)			(-0.86)
**FDI**	-1.378**	-1.395**	-1.334**	-2.019***	-2.049***	-1.946***	-0.644	-0.655	-0.64
	(-2.09)	(-2.14)	(-2.05)	(-3.11)	(-3.18)	(-3.00)	(-0.96)	(-0.99)	(-1.00)
**LnME**	-0.027**	-0.024*	-0.020	-0.014	-0.012	-0.012	-0.043***	-0.039***	-0.040***
	(-2.00)	(-1.76)	(-1.48)	(-1.12)	(-0.95)	(-0.93)	(-3.16)	(-2.91)	(-2.94)
**EN**	0.550***	0.563***	0.552***	0.559***	0.549***	0.548***	0.382**	0.375**	0.370**
	(2.77)	(2.86)	(2.82)	(3.26)	(3.24)	(3.24)	(2.06)	(2.05)	(2.02)
**INS**	0.350	0.320	0.317	-1.382**	-1.369**	-1.384**	0.805	0.748	0.759
	(0.55)	(0.51)	(0.50)	(-2.42	(-2.42)	(-2.45)	(1.55)	(1.45)	(1.47)
**URB**	2.228***	2.179***	2.131***	1.944***	1.953***	1.916***	2.979***	2.963***	2.929***
	(5.35)	(5.27)	(5.17)	(4.35)	(4.41)	(4.32)	(6.69)	(6.73)	(6.63)
**W-TFP**	0.072**	0.182	-0.255	0.054	0.134	-0.109	0.006	-0.044	-0.081
	(1.96)	(1.44)	(0.85)	(0.96)	(0.87)	(-0.28)	(0.19)	(-0.58)	(-0.42)
**W-TFP** ^**2**^		-0.025	0.221		-0.018	0.114		0.013	0.027
		(-0.96)	(1.42)		(-0.52)	(0.58)		(0.74)	(0.37)
**W-TFP** ^**3**^			-0.022			-0.018			-0.003
			(-1.60)			(-0.69)			(-0.26)
**W-FDI**	-13.801***	-13.920***	-12.998***	-19.436**	-20.623***	-19.469***	-1.235	-1.197	-1.284
	(-3.42)	(-3.43)	(-3.19)	(-3.49)	(-3.73)	(-3.48)	(-0.48)	(-0.47)	(-0.50)
**W-lnME**	-0.086	-0.070	-0.063	0.202**	0.174**	0.180**	-0.170***	-0.156***	-0.155**
	(-1.10)	(-0.91)	(-0.81)	(2.30)	(1.99)	(2.05)	(-3.30)	(-3.04)	(-3.01)
**W-EN**	2.435**	2.714***	2.507**	4.503***	4.412***	4.402***	0.994	1.021	1.013
	(2.37)	(2.65)	(2.44)	(3.80)	(3.76)	(3.76)	(1.32)	(1.37)	(1.36)
**W-INS**	-3.679	-3.760	-3.483	-21.525***	-21.625***	-21.714***	-1.420	-2.064	-2.140
	(-1.01)	(-1.04)	(-0.97)	(-5.75)	(-5.84)	(-5.88)	(-0.77)	(-1.12)	(-1.16)
**W-URB**	-2.374	-2.616	-2.682	-0.992	-0.333	-0.446	2.813**	2.825**	2.776**
	(-0.77)	(-0.85)	(-0.88)	(-0.25)	(-0.09)	(-0.11)	(1.97)	(1.99)	(1.96)
**R** ^**2**^	0.286	0.282	0.266	0.242	0.220	0.226	0.350	0.363	0.346
**Log-L**	304.790	308.423	310.018	323.416	327.030	327.753	298.000	301.703	302.084
**obs**	330	330	330	330	330	330	330	330	330

Note: Figures in parentheses are Z-value.

***, **and * indicate the significance at 1%,5% and 10% levels respectively.

**Table 10:** Estimation results of SDM.

	(1)	(2)	(3)	(4)	(5)	(6)	(7)	(8)	(9)
	W_1_			W_2_			W_3_		
**W-lnCO**_**2**_	-0.464**	-4.588**	-0.465**	-0.746***	-0.736***	-0.791***	0.069	0.068	0.068
	(-2.42)	(-2.39)	(-2.42)	(-3.04)	(-2.99)	(-3.20)	(0.54)	(0.54)	(0.54)
**EC**	0.010	0.018	-0.128	0.0158	0.008	-0.188*	0.011	0.021	-0.121
	(0.87)	(0.44)	(-1.12)	(1.47)	(0.21)	(-1.68)	(0.95)	(0.52)	(-1.06)
**EC** ^**2**^		-0.003	0.099		0.002	0.140*		-0.003	0.095
		(-0.20)	(1.32)		(0.21)	(1.88)		(-0.27)	(1.25)
**EC** ^**3**^			-0.020			0.027*			-0.019
			(-1.37)			(-1.87)			(-1.31)
**FDI**	-1.373**	-1.342**	-1.278*	-1.912***	-1.923***	-1.973***	-0.561	-0.555	-0.517
	(-2.06)	(-2.01)	(-1.92)	(-2.91)	(-2.92)	(-3.03)	(-0.83)	(-0.82)	(-0.76)
**LnME**	-0.028**	-0.028**	-0.028**	-0.015	-0.015	-0.015	-0.044***	-0.044***	-0.044***
	(-2.05)	(-2.00)	(-1.99)	(-1.13)	(-1.13)	(-1.17)	(-3.22)	(-3.21)	(-3.19)
**EN**	0.571***	0.572***	0.553***	0.572***	0.578***	0.505***	0.394**	0.389**	0.390**
	(2.81)	(2.82)	(2.70)	(3.24)	(3.26)	(2.83)	(2.11)	(2.06)	(2.07)
**INS**	0.423	0.433	0.430	-1.437**	-1.4552**	-1.449**	0.734	0.753	0.749
	(0.65)	(0.67)	(0.66)	(-2.48)	(-2.50)	(-2.52)	(1.39)	(1.43)	(1.42)
**URB**	2.225***	2.236***	2.232***	1.929***	1.945***	1.878***	3.039***	3.023***	3.020***
	(5.28)	(5.31)	(5.31)	(4.25)	(4.27)	(4.16)	(6.73)	(6.68)	(6.62)
**W-EC**	0.108*	-0.017	-0.333	0.037	-0.108	-1.924**	-0.011	0.039	0.147
	(1.92)	(-0.07)	(-0.53)	(0.51)	(-0.38)	(-2.22)	(0.751)	(0.30)	(0.39)
**W-EC** ^**2**^		0.039	0.256		0.046	1.258**		-0.018	-0.087
		(0.55)	(0.61)		(0.53)	(2.29)		(-0.41)	(-0.33)
**W-EC** ^**3**^			-0.041			-0.238**			0.013
			(-0.52)			(-2.24)			(0.803)
**W-FDI**	-15.121***	-14.762***	-14.091***	-18.187***	-18,295***	-18.020***	-0.583	-0.608	-0.629
	(-3.75)	(-3.61)	(-0.52)	(-3.22)	(-3.23)	(-3.21)	(-0.22)	(-0.23)	(-0.24)
**W-lnME**	-0100	-0.103	-0.102	0.209**	0.211**	0.1991**	-0.176***	-0.176***	-0.176***
	(-1.28)	(-1.31)	(-1.30)	(2.33)	(2.35)	(2.24)	(-3.38)	(-3.38)	(-3.39)
**W-EN**	2.458**	2.282**	2.117*	4.530***	4.505***	4.375***	1.114	1.121	1.101
	(2.35)	(2.11)	(1.93)	(3.68)	(3.64)	(3.53)	(1.51)	(1.48)	(1.44)
**W-INS**	-2.789	-2.869	-2.568	-21.678***	-21.904***	-21.763***	-1.683	-1.545	-1.588
	(-0.75)	(-0.77)	(-0.69)	(-5.69)	(-5.71)	(-5.74)	(-0.90)	(-0.82)	(-0.84)
**W-URB**	-2.807	-2.601	-3.059	-1.683	-1.4670	-1.829	2.968**	2.885**	2.697*
	(-0.90)	(-0.83)	(-0.97)	(-0.42)	(-0.37)	(-0.46)	(2.05)	(1.97)	(1.84)
**R** ^**2**^	0.312	0.307	0.331	0.292	0.289	0.325	0.301	0.303	0.254
**Log-L**	301.733	301.938	302.883	318.480	318.626	321.615	294.635	294.50	295.703
**obs**	330	330	330	330	330	330	330	330	330

**Table 11:** Estimation results of SDM.

	(1)	(2)	(3)	(4)	(5)	(6)	(7)	(8)	(9)
	W_1_			W_2_			W_3_		
**W-lnCO**_**2**_	-0.515***	-0.495**	-0.512***	-0.791***	-0.778***	-0.819***	0.061	0.024	0.020
	(-2.64)	(-2.54)	(-2.60)	(-2.19)	(-3.16)	(-3.31)	(0.48)	(0.19)	(0.16)
**TC**	0.030**	-0.057*	-0.127	0.036***	-0.047	-0.193**	0.029**	-0.072**	-0.193**
	(2.20)	(-1.74)	(-1.43)	(2.71)	(-1.51)	(-2.13)	(2.14)	(-2.11)	(-2.00)
**TC** ^**2**^		0.020***	0.062		0.020***	0.100**		0.024***	0.088*
		(2.84)	(1.29)		(2.93)	(2.14)		(3.22)	(1.81)
**TC** ^**3**^			-0.005			-0.010*			0.008
			(-0.87)			(-1.73)			(-1.33)
**FDI**	-1.406**	-1.442**	-1.413**	-2.098***	-2.232***	-2.115***	-0.540	-0.706	-0.704
	(-2.13)	(-2.22)	(-2.17)	(-3.18)	(-3.41)	(-3.23)	(-0.80)	(-1.07)	(-1.07)
**LnME**	-0.029**	-0.024*	-0.022	-0.013	-0.009	-0.008	-0.042***	-0.036***	-0.036***
	(-2.09)	(-1.73)	(-1.62)	(-0.98)	(-0.70)	(-0.61)	(-3.10)	(-2.64)	(-2.62)
**EN**	0.471**	0.465**	0.469**	0.505***	0.481***	0.490***	0.368**	0.367**	0.387**
	(2.36)	(2.36)	(2.37)	(2.91)	(2.80)	(2.87)	(1.98)	(2.00)	(2.11)
**INS**	0.303	0.335	0.364	-1.432**	-1.348**	-1.230**	0.718	0.833	0.872*
	(0.47)	(0.53)	(0.57)	(-2.50)	(-2.37)	(-2.24)	(1.38)	(1.62)	(1.70)
**URB**	2.243***	2183***	2.163***	1.970***	1.960***	1.992***	3.059***	2.845***	2.804***
	(5.35)	(5.28)	(5.23)	(4.38)	(4.42)	(4.51)	(6.86)	(6.40)	(6.24)
**W-TC**	0.109*	0.234	-0.060	0.122	0.293	-0.550	0.035	-0.158	-0.247
	(1.70)	(1.28)	(-0.14)	(1.32)	(1.26)	(-0.74)	(0.67)	(-1.29)	(0.63)
**W-TC** ^**2**^		-0.027	0.120		-0.035	0.433		0.046*	0.095
		(-0.68)	(0.58)		(-0.75)	(1.10)		(1.72)	(0.47)
**W-TC** ^**3**^			-0.018			0.433			-0.006
			(-0.72)			(1.10)			(-0.24)
**W-FDI**	-14.624***	-13.753***	-13.337***	-20.850***	-22.715***	-21.366***	-0.777	-1.666	-1.741
	(-3.16)	(-3.41)	(-3.30)	(-3.68)	(-4.02)	(-3.77)	(-0.30)	(-0.65)	(-0.68)
**W-lnME**	-0.102	-0.111	-0.100	0.205**	0.161*	0.179**	-0.176***	-0.151***	-0.143***
	(-1.31)	(-1.44)	(-1.28)	(2.31)	(1.82)	(2.01)	(-3.41)	(-2.93)	(-2.74)
**W-EN**	1.979*	2.217**	2.162**	3.846***	3.830***	3.830***	0.969	0.894	0.915
	(1.94)	(2.19)	(2.14)	(3.20)	(3.21)	(3.22)	(1.28)	(1.19)	(1.21)
**W-INS**	-4.419	-4.837	-4.547	-21.663***	-2.032***	-21.320***	-1.743	-1.588	-1.706
	(-1.21)	(-1.34)	(-1.26)	(-5.78)	(-5.90)	(-5.69)	(-0.94)	(-0.87)	(-0.94)
**W-URB**	-2.149	-2.086	-2.295	-1.040	0.233	0.643	3.138**	2.476*	2.258
	(-0.69)	(-0.68)	(-0.74)	(-0.26)	(0.06)	(0.17)	(2.18)	(1.72)	(1.56)
**R** ^**2**^	0.315	0.318	0.313	0.243	0.200	0.140	0.364	0.383	0.330
**Log-L**	302.835	307.679	308.234	321.089	329.002	327.685	296.554	302.057	302.946
**obs**	330	330	330	330	330	330	330	330	330

Other control variables also have certain effect on CO_2_ emissions. According to Tables 9–11, in most models, the estimation coefficients of FDI are negative, and they all pass the significance test at 10% significance level, this indicates that the increase of FDI will significantly reduce the increase of CO_2_ emissions. For example, according to the column (3) of Table 9, every 1% increase in FDI, CO_2_ emissions will decrease by 1.334%. By comparing Tables 9–11, it is not difficult to find that the estimated coefficients of EN under the three weight matrices are significantly positive at 1% statistical level, which can fully demonstrate energy consumption can damage the environment. For every 1% increase in energy consumption, CO_2_ emissions increase by about 0.35%-0.55%, which indicates that controlling energy consumption and producing clean energy is an important part to promote China’s sustainable development. Similarly, under the three weight matrices, the estimated coefficients of URB are consistent, they are all significant at 1% significance level. According to the column (3) in Table 9, for example, if URB increases by 1%, CO_2_ emissions will increase by about 2.13%. In addition, the impact of lnME on CO_2_ emissions are all negative in most models, and most models pass the significance test at 10% significance level. This may be because China has gradually got rid of its excessive dependence on energy consumption in the stage of technology research and development, and has created a benign environment for innovation, obviously, the vibrant innovation market environment will significantly reduce the regional CO_2_ emissions. Finally, the estimated coefficients of INS is not significant in most models.

From Table 9, the TFP of high-tech industries not only affects CO_2_ emissions in local region, also affects CO_2_ emissions in neighboring regions, and it also presents a characteristic of inverted “N” shaped curve. However, the spatial spillover effects of the first, quadratic and cubic term of TFP are not significant. It should note that the spatial effects of EC and TC are also similar to TFP. By taking the Eq (6) of Table 11 as example, the coefficients of spatial lag term of the first and cubic term of EC are -1.924、-0.238 respectively, the coefficient of the quadratic term is 1.258, and they all pass the 5% significance test. From Tables 9–11, the coefficients of the spatial lag term of FDI are all negative and pass the significance test in most models, which means the CO_2_ emissions in one region are only affected by local FDI, but also by FDI from other regions. Because of the “radiation effect” and “technology spillover effect” among each province, adjacent regions have similar foreign investment environment, and the increase of FDI in adjacent regions will also drive the development of FDI in local region, thus reducing CO_2_ emissions. In addition, the spatial lag term coefficients of EN and FDI is opposite, it shows a positive spatial spillover effect, in other words, the increase of EN in adjacent regions will promote the increase of CO2 emissions in local region.

### Direct effect, indirect effect and total effect

For further analyzing the spatial effect of TFP in high-tech industries on CO_2_ emissions, this paper analyzes the effect of TFP and other factors on CO_2_ emissions through the analysis of direct effect, indirect effect and total effect under the full sample. The result is shown in Table 12, it is obvious that the estimation coefficients of direct effect, indirect effect and total effect of the first and cubic term of TFP are all negative, and the estimation coefficients of quadratic term is positive, and they all pass the significance test at 10% significance level. The direct, indirect and total effect of the first, quadratic and cubic terms of EC and TC show the same characteristics as TFP, however, the direct effect of EC and TC are not significant. This can be caused by the industrial characteristics of high-tech industry. Due to its intelligence and innovation as well as intensive industrial knowledge and technology, the proportion of scientific and technological personnel is relatively high, therefore, it is difficult to carry out the flow of technology and human resources between regions, and the scale efficiency has a greater impact on neighboring regions.

**Table 12:** Direct effect, indirect effect and total effect.

	Direct effect			Indirect effect			Total effect		
**TFP**	-0.149**			-0.484**			-0.633***		
	(-2.21)			(-2.51)			(-2.64)		
**TFP** ^**2**^	0.073**			0.318***			0.391***		
	(2.15)			(2.67)			(3.09)		
**TFP** ^**3**^	-0.008*			-0.044***			-0.052***		
	(-1.73)			(-2.75)			(-3.03)		
**EC**		-0.170			-1.173**			-1.343***	
		(-1.33)			(-2.41)			(-2.64)	
**EC** ^**2**^		0.124			0.767**			0.890***	
		(1.47)			(2.36)			(2.63)	
**EC** ^**3**^		-0.239			-0.130**			-0.154**	
		(-1.49)			(-2.08)			(-2.36)	
**TC**			-0.176			-0.348			-0.524
			(-1.63)			(-1.01)			(-1.46)
**TC** ^**2**^			0.081			0.257			0.337**
			(1.49)			(1.57)			(1.99)
**TC** ^**3**^			-0.007			-0.034*			-0.041**
			(-1.06)			(-1.68)			(-1.96)

Note: Figures in parentheses are Z-value.

***, **and * indicate the significance at 1%,5% and 10% levels respectively.

### Robust check

This paper uses industrial solid waste discharges to replace the CO_2_ emissions to perform a robust check, and it finds the empirical results are similar. All of the results are presented in the Table 13. The research finds that the industrial solid waste discharges also have significant negative spatial spillover effect, as shown in Table 13 (1), for example, for every 1% increase in industrial solid waste discharges in the local region, solid waste discharges in neighboring regions are reduced by nearly 1%. The effect of TFP, EC and TC in high-tech industries on industrial solid wasted discharges also shows a nonlinear inverted “N” shape, however, only EC passes the significance test. In addition, the regression results of other control variables are also basically consistent with the above results.

**Table 13:** Robustness check of SDM.

	(1)	(2)	(3)	(4)	(5)	(6)	(7)	(8)	(9)
	W_1_			W_2_			W_3_		
**W-lnSolid**	-0.999***	-0.986***	-1.015***	-0.983***	-1.005***	-1.041***	-0.983***	-0.986***	-0.984***
	(-7.10)	(-7.08)	(-7.23)	(-7.20)	(-7.37)	(-7.69)	(-7.02)	(-7.04)	(-7.00)
**TFP**	0.025	-0.007	-0.144						
	(1.42)	(-0.14)	(-1.15)						
**TFP** ^**2**^		0.008	0.082						
		(0.71)	(1.28)						
**TFP** ^**3**^			-0.010						
			(-1.18)						
**EC**				0.007	-0.110	-0.552**			
				(0.32)	(-1.34)	(-2.42)			
**EC** ^**2**^					0.038	0.335**			
					(1.50)	(2.30)			
**EC** ^**3**^						-0.059**			
						(-2.08)			
**TC**							0.059**	0.015	-0.279
							(2.17)	(0.23)	(-1.43)
**TC** ^**2**^								0.011	0.166*
								(0.72)	(1.69)
**TC** ^**3**^									-0.020
									(-1.59)
**FDI**	-4.677***	-4.709***	-4.605***	-4.618***	-4.582***	-4.306***	-4.667***	-4.620***	-4.546***
	(-3.49)	(-3.51)	(-3.44)	(-3.44)	(-3.42)	(-3.25)	(-3.50)	(-3.47)	(-3.42)
**LnME**	-0.114***	-0.112***	-0.106***	-0.114***	-0.107***	-0.108***	-0.113***	-0.113***	-0.110***
	(-4.09)	(-3.99)	(-3.75)	(-4.07)	(-3.83)	(-3.92)	(-4.08)	(-4.05)	(-3.93)
**EN**	1.471***	1.480***	1.467***	1.511***	1.521***	1.366***	1.377***	1.401***	1.427***
	(3.61)	(3.64)	(3.62)	(3.67)	(3.71)	(3.34)	(3.41)	(3.46)	(3.53)
**INS**	-0.018	-0.029	-0.042	0.072	0.046	0.50	-0.051	0.004	0.0754
	(-0.01)	(-0.02)	(-0.03)	(0.05)	(0.03)	(0.12)	(-0.04)	(0.00)	(0.06)
**URB**	4.843***	4.795***	4.706***	4.842***	4.906***	4.819***	4.877***	4.890***	4.845***
	(5.70)	(5.63)	(5.53)	(5.68)	(5.78)	(5.75)	(5.76)	(5.77)	(5.74)
**W-TFP**	0.049	0.179	-0.543						
	(0.65)	(0.69)	(-0.88)						
**W-TFP** ^**2**^		-0.029	0.377						
		(-0.54)	(1.17)						
**W-TFP** ^**3**^			-0.055						
			(-1.27)						
**W-EC**				0.129	-0.421	-3.318***			
				(1.13)	(-0.89)	(-2.63)			
**W-EC** ^**2**^					0.174	2.192***			
					(1.22)	(2.63)			
**W-EC** ^**3**^						-0.389**			
						(-2.44)			
**W-TC**							0.078	-0.226	-.762
							(0.59)	(-0.60)	(-0.86)
**W-TC** ^**2**^								0.071	0.332
								(0.382)	(0.79)
**W-TC** ^**3**^									0.332
									(0.79)
**W-FDI**	-46.943***	-47.331***	-45.841***	-49.122***	-46.566***	-41.770***	-47.607***	-46.579***	-45.495***
	(-5.72)	(-5.69)	(-5.48)	(-5.91)	(-5.69)	(-5.04)	(-5.83)	(-5.68)	(-5.52)
**W-lnME**	-0.809***	-0.798***	-0.780***	-0.833***	-0.807***	-0.807***	-0.823***	-0.820***	-0.771***
	(-5.22)	(-5.14)	(-4.96)	(-5.37)	(-5.18)	(-5.25)	(-5.36)	(-5.34)	(-0.64)
**W-EN**	3.260	3.491	3.261	3.444	3.331	2.373	2.663	2.755	2.486
	(1.48)	(1.58)	(1.48)	(1.56)	(1.48)	(1.05)	(1.23)	(1.27)	(1.14)
**W-INS**	-5.587	-5.528	-5.133	-4.203	-4.356	-2.177	-6.068	-5.403	-4.749
	(-0.75)	(-0.74)	(-0.69)	(-0.56)	(-0.58)	(-0.29)	(-0.82)	(-0.73)	(-0.64)
**W-URB**	8.151	7.890	7.648	7.905	8594	6.147	8.333	7.976	6.942
	(1.33)	(1.28)	(1.25)	(1.28)	(1.39)	(1.00)	(1.36)	(1.30)	(1.13)
**R** ^**2**^	0.162	0.162	0.160	0.158	0.161	0.155	0.161	0.161	0.162
**Log-L**	63.151	63.661	64.770	62.554	64.064	68.036	64.249	64.780	66.129
**obs**	330	330	330	330	330	330	330	330	330

Note: Figures in parentheses are Z-value.

***, **and * indicate the significance at 1%,5% and 10% levels respectively.

Then we make further predictions. Considering the time delay, we only introduce TFP and the quadratic term of TFP into the model, and use the spatial econometric model regression method above for further regression. The results are shown in Table 14 below.

**Table 14:** Robustness check.

SDM			
W-lnCO2	-0.430***(-2.64)		
**TFP**	0.510***(3.38)	W-TFP	0.100(1.03)
**TFP** ^**2**^	-0.073***(-3.75)	W-TFP2	-0.032**(-2.03)
**FDI**	0.118**(2.48)	W-FDI	-0.332(-0.92)
**LnME**	0.002*(1.92)	W-lnME	0.026***(4.64)
**EN**	0.005(0.34)	W-EN	0.272***(3.72)
**INS**	-0.044(-1.03)	W-INS	0.414*(1.67)
**URB**	-0.076*(-1.78)	W-URB	0.253(1.03)
**R** ^**2**^	0.835	obs	330

Note: Figures in parentheses are Z-value.

***, **and * indicate the significance at 1%,5% and 10% levels respectively.

We find that the regression results still show the role of promotion first and then inhibition, and the inhibition effect is enhanced, that is, the innovation efficiency of high-tech industry has an inhibitory effect on CO_2_ emission, and this inhibitory effect is gradually enhanced. The robustness of the above results is proved again.

## Discussion

Based on the above empirical analysis, this paper obtains three interesting findings. First of all, CO_2_ emissions in various regions of China are highly unbalanced, and it shows a decreasing trend from eastern coastal region to middle and western region [62]. The region with high emissions are mainly concentrated in the eastern coastal area, especially the Bohai Rim region and Yangtze River Delta region [1]. In addition, CO_2_ emissions have significant negative spatial spillover effect, which means CO_2_ emissions from adjacent areas will reduce CO_2_ emissions from the local region. In recent years, China is changing from “extensive economic growth mode” to “intensive economic growth mode, therefore, economic growth is becoming less dependent on energy consumption, and due to the existence of “warning effect” among provinces, if the neighboring province promotes the economic development at the expense of environment, it will alert the local region to control the usage of energy, and thus reducing CO_2_ emissions. However, if adding innovation factor to the spatial weight matrix, the opposite result will be obtained. This also indicates that there may be a phenomenon of “blind competition” in the pursuit of technical innovation and development, that is, formed a “Bandwagon Effect”.

Secondly, in a spatial perspective, the effect of innovation efficiency in high-tech industries on CO_2_ emission is not a simple linear relationship. Xu and Lin(2017) also comes to a similar conclusion, they pointed out that the nonlinear model will obtain a better fitting effect than the traditional linear regression model when analyzing the effect of innovation efficiency in high-tech industries on CO_2_ emissions [3]. However, they only add quadratic term into their analysis, this paper further adds cubic term for conducting regression. According to the above results, this paper finds that the coefficients of the first-order term and the cubic term of TFP are negative, while the coefficients of the quadratic term are positive, which indicates CO_2_ emissions present an “inverted N shape” curve with the growth of TFP. In other words, in the early stage, the improvement of TFP in high-tech industries will reduce the regional CO_2_ emissions, however, with the further study on innovation, innovation input will significantly increase, but due to the innovative achievement exists certain lag, CO_2_ emissions will increase with the rise of TFP. In the later stage, the innovation achievements of the previous stage have been preliminarily formed, and the inhibiting effect of TFP on CO_2_ emissions starts to appear, however, from the current view, it still has a long way to reach the target. Moreover, the regression results of TC and EC is consistent with the results of TFP, especially the effect of TC on CO_2_ emissions is more significant, and it also proves the improvement of technical level is an important way to conserve energy and reduce emissions. This is consistent with the classical Environment Kuznets (EKC) Hypothesis, however, EKC hypothesis also pointed out that there is a decoupling stage between environmental pollution and economic growth, but the timing of this stage is ambiguous, which also demonstrates the difficulty of environmental governance.

Thirdly, foreign direct investment (FDI) and urbanization level (URB) will also affect regional CO_2_ emissions. In terms of FDI, it will significantly reduce CO_2_ emissions. On the one hand, due to FDI has brought advanced technology and talents to the local region, and it may reduce CO_2_ emissions by technical means. On the other hand, foreign invested enterprises should conform to the strict pollution regulation of foreign countries, which also requires the local region to strengthen the environment management, so as to fully utilize the positive technical spillover advantage of FDI. In terms of URB, it will promote the increase of CO_2_ emissions. On the one hand, due to the “scale effect”, the improvement of urbanization level will produce a large number of housing demand, increase household electricity consumption and demand for motor vehicles, and these three factors will significantly increase CO_2_ emissions. Moreover, traffic congestion and high density of residents are not conducive to the full combustion and diffusion of automotive fuels [63]. On the other hand, due to the “agglomeration effect”, the improvement of urbanization level will bring the aggregation of population, thus enhancing the service efficiency of resources and share ratio of public facilities, and alleviate the environmental harm brought by CO_2_ emissions [64]. According to the results in Table 8, “scale effect” is significantly greater than “agglomeration effect”, it also demonstrates that the government should give full play to the positive role of “agglomeration effect” in urban construction.

## Conclusion and policy recommendations

To sum up, this paper, based on a spatial perspective, uses the provincial panel data in China from 2006–2016 as the research object, and it explores whether the CO_2_ emissions exit spatial spillover effect. On this basis, it constructs three different weight matrices (spatial geography, spatial economic geography nesting and innovation) and uses the spatial econometric model to empirical analyze the effect of innovation efficiency and its decomposition index in high-tech industries on CO_2_ emissions. Then, it also further analyzes its direct effect, indirect effect and total effect. Finally, for ensuring the robustness of the results, this paper also uses industrial solid waste discharges to replace CO_2_ emissions to conduct spatial regression again. This paper finds: (1) from 2006 to 2012, the CO_2_ emissions in China increase with years, however, after 2012, the CO_2_ emissions in some provinces experience a decline with different degrees. In addition, the CO_2_ emissions present a decreasing trend from eastern coastal area to central and western regions. (2) The innovation efficiency of high-tech industries (TFP) shows an upward trend in general, however, the overall level is not high, and its decomposition index, technical progress (TC) is the major factor to effect the change of TFP in high-tech industries. (3) From the regression results of panel data, the coefficients of the first-order term and cubic term of innovation efficiency in China’s high-tech industries are negative, the coefficients of the quadratic terms are positive, however, the coefficients of the cubic term are not significant. In other words, the effect of TFP in high-tech industries on CO_2_ emissions is not a simple linear relationship, but shows a trend of “inverted N-shaped” curve, and the effect of decomposition index, changes in technical efficiency (EC) and efficiency of technical progress (TC), on CO_2_ emissions is similar to TFP. (4) CO2 emissions in various regions of China are highly unbalanced, and due to the existence of “warning effect”, CO2 emissions in various regions have significant negative spatial spillover effect.

According to the above conclusions, this paper proposes the following recommendations:

1. The government should vigorously promote the development of high-tech industries and improve the innovation efficiency. In the long run, the improvement of innovation efficiency in high-tech industries will reduce CO_2_ emissions, accelerate the formation of “low-carbon economy” and promote sustainable development. Therefore, it is crucial to promote the development of high-tech industries, especially the promotion of high-tech industries, such as new energy or environmental protection. However, at present, the driving force for the development of new energy high-tech industries is insufficient, the market safeguard mechanism is not well developed. Thus, for the government, it should improve the market safeguard mechanism of new energy high-tech industry, guide and encourage the social capital make investment to new energy technology industry, especially the Green investment. At the same time, the government also need to provide certain economic policies to incentive these investments. In addition, for the traditional industry with high CO_2_ emissions, the government should encourage its transformation through the means of subsidies and others to reduce CO_2_ emissions.
2. The government should optimize the development environment of innovation in high-tech industries and improve the internal management of high-tech industries. According to the regression result of EC, it is easy to find that the improvement of technical efficiency also plays a positive role in energy conservation and emission reduction. This will require the high-tech industry to optimize its internal environment and conduct effective management. China has a vast territory and abundant resources, therefore, each region with different factor endowments should adjust measures to local conditions and give full play to their own regional characteristics. For the eastern region with well-developed industries, they need to take advantage of location and talent, while for the middle and western region, the government should increase investment in research and development (R&D) and training expenditure on R&D personnel. The high-tech industry, as a knowledge-intensive industry, has a higher requirement for R&D personnel. Therefore, it should pay more attention to the recruitment and training of certain majors, such as biological science, marine science, ecology, and environmental science when conducting the personnel training of higher education.
3. The government should increase investment in basic R&D input related to high-tech industries and promote technical progress in these industries. On the one hand, the government should show its leading role in technological innovation, guide the ideas of independent innovation, and improve the incentive and assessment mechanism for effective invention patents, and stimulate the vitality of original technological innovation. Simultaneously, the government should also remove the technical barriers in each region to accelerate the improvement of the overall technical starting point. On the other hand, the local government may also encourage enterprises to introduce advanced technologies from other regions by cutting taxes or providing low-interest loans, thus it can fully utilize the positive externalities of technological innovation.
